# Craniofacial fractures sustained under the influence of alcohol: what are the differences between the sexes?

**DOI:** 10.2340/aos.v83.41381

**Published:** 2024-09-09

**Authors:** Hanna Thorén, Klaus Virtanen, Erkka Oksanen, Miika Toivari, Auli Suominen, Tero Puolakkainen, Johanna Snäll

**Affiliations:** a Department of Oral and Maxillofacial Surgery, Institute of Dentistry, University of Turku, Turku, Finland; b Department of Oral and Maxillofacial Diseases, University of Helsinki and Helsinki University Hospital, Helsinki, Finland; c Department of Community Dentistry, Institute of Dentistry, University of Turku, Turku, Finland

**Keywords:** Facial fracture, trauma, alcohol, intoxication

## Abstract

**Objective:**

To identify mechanisms and types of injuries in patients having sustained craniofacial fractures under the influence of alcohol, and to compare the frequencies of them between males and females.

**Materials and methods:**

Patients included were adults who had been diagnosed with craniofacial fractures at Töölö Hospital Emergency Department, Helsinki University Hospital, Finland, and who had been under the influence of alcohol at the time of injury. The primary outcome variables were assault-related and fall-related injury mechanisms. The secondary outcome variables were other injury mechanisms, time of accident, type of craniofacial fracture and severity of facial fracture. The primary predictor variable was sex. The control variable was age at the time of injury. The statistical modelling was executed using logistic regression.

**Results:**

Of the total of 2,859 patients with craniofacial fractures, 1,014 patients (35.5%) fulfilled the inclusion criteria. Males predominated (84.6%). Assault (38.0%) was the most frequent aetiology. Compared to the odds of females, males had 2.8 times greater odds for assault, 2.4 times greater odds for isolated cranial fracture and 1.7 times greater odds for a facial injury severity score of ≥ 3. Females had 2.0 times greater odds for any fall compared to the odds of males.

**Conclusions:**

Particularly male patients are frequently under the influence of alcohol at the time of injury, predisposing them to assault and severe facial fractures more often than females. Codes of practice on how to identify unhealthy alcohol use and how to intervene are recommended.

## Introduction

Alcohol use is a major risk factor for diseases and injuries, causing a notable burden on health care and social assistance systems. Regarding injuries, it has been shown that consuming alcohol during 6 hours before being injured increased the odds for severe injury over 2-fold compared to not having consumed alcohol during the previous day [1]. A recently published study estimated that on average 15% (range 5–40%) of all injuries presenting at emergency departments across 27 countries were attributable to alcohol use [2].

Alcohol is a notable risk factor for facial injuries, as highlighted in several studies. Papers from the United States [3,4], the United Kingdom [5], France [6], Australia [7], Brazil [8] and Finland [9,10] reveal that alcohol was involved in one way or another in 18–55% of the patients. The corresponding rate is notable also among elderly patients, being 20.5% in patients aged 60 years or more [10] and 11.0% in those aged 65 years or more [11]. Moreover, it is well known from experience that regular heavy alcohol use predisposes for recurrent facial injuries.

Men predominate clearly (85–88%) among patients having sustained facial injuries under the influence of alcohol [7,9]. The finding is likely attributable to the differences in alcohol use between men and women. Worldwide, alcohol use as well as high-volume drinking is more prevalent among men [12]. However, as far as we know, studies focussing on alcohol-related mechanisms of craniofacial fractures in general, and differences in these mechanisms between men and women in particular, are scarce.

The aim of the present study was to investigate the characteristics of patients having sustained craniofacial fractures under the influence of alcohol. The specific aims were to identify trauma mechanisms, types and severities of fractures, and to compare the frequencies of them between males and females.

## Materials and methods

### Study design and sample description

To address the research aims, a retrospective cohort study was designed and implemented. During the 6-year period 2013–2018, a total of 2,859 patients aged 20 years or more had been diagnosed with craniofacial fractures (i.e. fractures of any bones in the face, skull vault or skull base) at a level I trauma centre (Töölö Hospital Emergency Department, Helsinki University Hospital, Finland). Included in the present study were those patients who had been under the influence of alcohol at the time of the injury. Patients were included if at least one of the following information items could be obtained from their patient file: (1) the patient had been tested positive for breath or blood alcohol at admission, (2) the patient had been identified as being under the influence of alcohol by breath odour at admission, or (3) in cases of delayed admission, the patient had informed the emergency department staff of having been under the influence of alcohol at the time of the injury.

## Descriptive statistics

Descriptive statistics were calculated for all variables included in the data analysis. Additional descriptive statistics with percentage values were presented for associations between sex and age groups (at intervals of 10-years), between sex and type of craniofacial fracture in assaulted patients, between sex and type of craniofacial fracture in patients having sustained their injuries by any fall, and between energy of trauma mechanism and type of craniofacial fracture.

### Data analyses

The primary predictor variable was sex.

The primary outcome variables were presence or absence of trauma mechanisms related to assault and any fall (including fall on the ground, fall from stairs and fall from height).

The secondary outcome variables were other trauma mechanisms, day of the week at the time of the accident, season at the time of the accident, type of craniofacial fracture and facial injury severity score (FISS).

Other trauma mechanisms were grouped as follows: fall at ground level, fall from stairs, fall from height, bicycle accidents, motor vehicle accidents and struck by object. In addition, patients who had sustained injuries from high energy trauma mechanisms (including falls from height, falls on stairs and motor vehicle accidents) were identified.

Day of the week at the time of the accident was categorised as weekday (Monday through Thursday) and weekend (Friday through Sunday). Season at the time of the accident was classified as winter (December through February), spring (March through May), summer (June through August) and fall (September through November).

Type of craniofacial fracture was classified into three categories: (1) isolated facial fracture (i.e. one or more fractures of the mandible or the midface), (2) isolated cranial fracture (i.e. one or more fractures of the orbital roof, frontal bone, posterior wall of the frontal sinus, other bones of the cranial vault and skull base), and (3) combined facial and cranial fracture (i.e. any combinations of the aforementioned).

Facial injury severity score was calculated for each patient according to Bagheri et al. [13], slightly modified (Table 1), and further classified into two categories ≤ 2 and ≥3. Facial injury severity score was calculated for patients who had fractures of any bones in the mandible, midface or upper facial third (i.e. the orbital roof, frontal bone and posterior wall of the frontal sinus).

**Table 1:** Facial injury severity scale*.

Fracture site/type	Points
**Mandible**	
Dentoalveolar	1
Each fracture of symphysis/body/angle/ramus	2
Each fracture of condyle/coronoid	1
**Midface**	
Dentoalveolar	1
Maxillary sinus (not involved in other complex)	1
Zygomatico-orbital complex	1
Orbital floor ± medial wall (not involved in other complex)	1
Nasal (not involved in other complex)	1
Le Fort I	2
Le Fort II	4
Le Fort III	6
(Unilateral Le Fort fractures are assigned half the value)	
Naso-orbito-ethmoid-complex	3
**Upper face**	
Orbital roof/rim	1
Frontal bone	2
Posterior wall of frontal sinus	2

* Modified from Bagheri et al. [13].

The control variable was age at the time of the injury. Based on the median age of the patient cohort, age at the time of the injury was classified into two categories as ≤ 42.35 and ≥ 42.36 years.

The Pearson Chi-square test and Mann-Whitney U-test were used to determine the associations between the control variable and primary predictor, and between the control variable and primary outcomes. The risk ratio was calculated between primary predictor and primary outcomes. The statistical modelling was executed using logistic regression. Odds ratio (OR) with 95% confidence intervals (CIs) were calculated to examine the associations between primary predictors and primary and secondary outcomes. The association between primary predictor and primary outcomes was evaluated using multivariable logistic regression. The control variable was included in the multivariable model if the control variable was associated with the primary predictor and primary outcome alike with *p* < 0.05. Data analysis was performed using Statistical Package for the Social Sciences (SPSS) software (IBM SPSS v27.0, IBM Corp., Armonk, NY, USA). *p* < 0.05 was set as the threshold for statistical significance.

### Ethical approval

The Internal Review Board of the Head and Neck Center of the Helsinki University Hospital, Helsinki, Finland approved the study. Patient consent was not required because of the retrospective nature of the study.

## Results

### Descriptive statistics

Of the total of 2,859 patients aged 20 years or above with craniofacial fractures, 1,014 patients (35.5%) fulfilled the inclusion criteria of having sustained their injuries under the influence of alcohol. Table 2 shows baseline characteristics of the 1,014 patients. Males predominated (84.6%). The age range was 20.0–86.3 years (mean 44.1 years). Assault (38%) and fall at ground level (29.9%) were the most common mechanisms of injury. When all different types of falls were grouped together, any fall remained the single most frequent aetiology (42.1%). High energy trauma mechanisms occurred in 15.7% of the patients. Most injuries occurred during weekends (67.3%), and during summer (31.0%) and fall (27.4%). The most common fracture type was isolated facial fracture (74%). Facial injury severity score was calculated for 893 patients who had fractures of any bones in the mandible, midface or upper facial third. In these patients, the FISS range was 1–15 (mean 2.1), with the majority of patients having a score of ≤ 2 (72.2%).

**Table 2:** Baseline characteristics of 1,014 patients with craniofacial fractures sustained under the influence of alcohol.

	Number of patients	% of 1,014	% of 893
**Gender**			
Male	858	84.6	
Female	156	15.4	
**Age (years)**			
Range 20.0–86.3			
Mean 44.1			
Median 42.3			
**Age group (years)**			
≤ 42.35	507	50.0	
≥ 42.36	507	50.0	
**Trauma mechanism**			
Assault	385	38.0	
Fall at ground level	303	29.9	
Bicycle	114	11.2	
Fall in stairs	76	7.5	
Fall from height	48	4.7	
Unknown	44	4.3	
Motor vehicle accident	35	3.5	
Struck by object	9	0.9	
**Any fall**			
Yes	427	42.1	
**High energy trauma mechanism**			
Yes	159	15.7	
**Day of the week of accident**			
Weekday (Monday–Thursday)	332	32.7	
Weekend (Friday–Sunday)	682	67.3	
**Season of accident**			
Winter	203	20.0	
Spring	219	21.6	
Summer	314	31.0	
Fall	278	27.4	
**Type of craniofacial fractures**			
Isolated facial fracture	750	74.0	
Isolated cranial fracture	156	15.4	
Combined facial and cranial fracture	108	10.7	
**FISS**			
Range 1–15			
Mean 2.1			
Median 1			
≤ 2	645		72.2
≥ 3	248		27.8

Isolated facial fracture: mandible/midface fracture; Isolated cranial fracture: frontal bone/orbital roof/skull vault/skull base fracture.

FISS: facial injury severity score, calculated in 893 patients with mandible, midfacial and/or upper facial third fractures.

**Figure 1:**
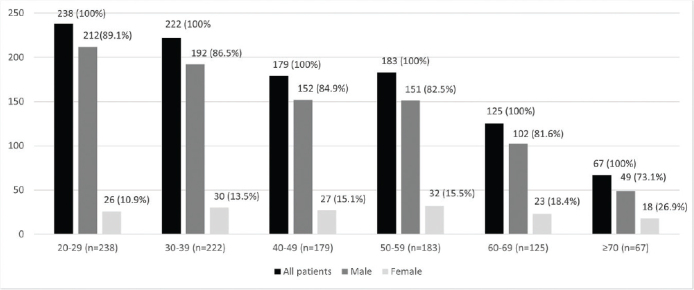
Association between sex and age groups.

Figure 1 describes the association between sex and age groups. There was a male preponderance in all age groups, the proportion decreasing with increasing age from 89.1% in the youngest age group to 73.1% in the oldest.

**Figure 2:**
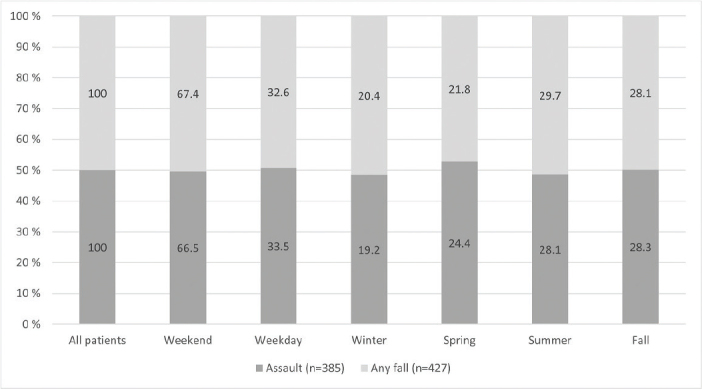
Distribution of assault and any fall by day of the week and season of the year.

Figure 2 shows the distribution of the two most common trauma mechanisms, assault and any fall, by day of the week and season of the year. Most assault related injuries (66.5%) as well as most injuries caused by any fall (67.4%) occurred during weekends. There were no notable differences in the rates of assaults and any falls when the four seasons were compared to each other.

**Figure 3:**
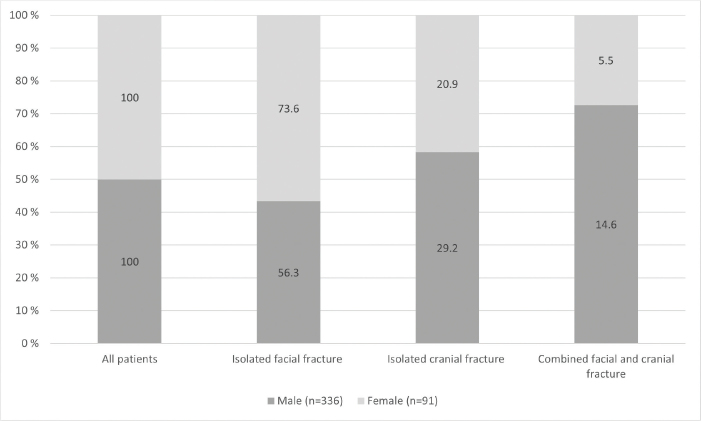
Association between sex and type of craniofacial fracture in patients who sustained their injury by any fall.

Figure 3 describes the association between sex and type of craniofacial fracture in patients who sustained their injury by any fall. In males and females alike, isolated facial fractures were most frequent, and combined facial and cranial fractures most infrequent. However, the proportion of isolated facial fractures was higher in females (73.6%) than in males (56.3%), whereas the proportion of combined craniofacial fractures was higher in males (14.6%) than among females (5.5%).

**Figure 4:**
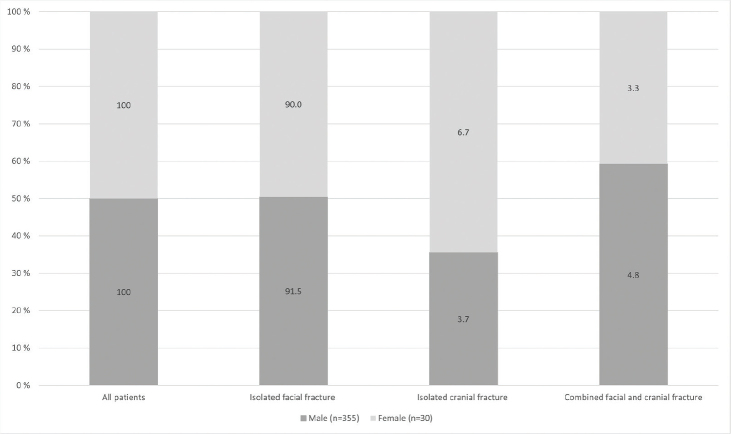
Association between sex and type of craniofacial fracture in assaulted patients.

Figure 4 describes the association between sex and type of craniofacial fracture in assaulted patients. Isolated facial fractures were, by far, most frequent in males (91.5%) as well as in females (90.0%).

**Figure 5:**
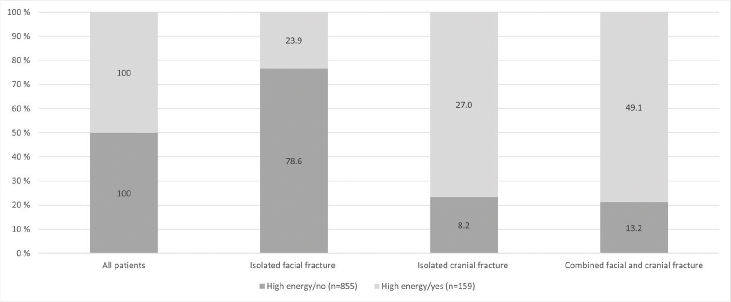
Association between energy of trauma and type of craniofacial fracture.

As shown in Figure 5, a high energy trauma mechanism most frequently caused combined facial and cranial fractures (49.1%) whereas mechanisms that were not because of high energy most often caused isolated facial fractures (78.6%).

### Data analysis

As shown in Tables 3 and 4, age was statistically significantly associated with sex (Table 2) and primary outcomes (Table 3). Mean age was significantly higher among females than males (*p* < 0.001), significantly higher in association with any fall as compared to no fall (*p* < 0.001), and significantly lower in patients who sustained their injuries due to assault as compared to those who did not (*p* < 0.001).

**Table 3:** Control variables by primary predictors.

	Male (*n* = 858)			Female (*n* = 156)			*P*
	Number of patients	% of 858		Number of patients	% of 156		
**Age (years)**							
Range			20.0–86.3			21.2–81.8	
Mean			43.4			48.1	< 0.001*
**Age group (years)**							0.005**
≤ 42.35	445	51.9		62	39.7		
≥ 42.36	413	48.1		94	60.3		

* Mann-Whitney U-test.

** Chi square.

**Table 4:** Control variables by primary outcomes.

	Assault/yes			Assault/no			*P*	Any fall/yes			Any fall/no			*P*
	Number of patients	% of *n*		Number of patients	% of *n*			Number of patients	% of *n*		Number of patients	% of *n*		
**All patients (*n* = 1,014)**	385	38.0		629	62.0			427	42.1		587	57.9		
**Age (years)**							< 0.001*							< 0.001*
Range			20.0–69.5			20.0–86.3				20.0–86.3			20.0–75.7	
Mean			35.9			49.2				51.2			39.0	
**Age group (years)**							< 0.001**							< 0.001**
≤ 42.35 (*n* = 507)	278	54.8		229	45.2			136	26.8		371	73.2		
≥ 42.36 (*n* = 507)	107	21.1		400	78.9			291	57.4		216	42.6		

* Mann-Whitney U-test.

** Chi square.

Table 5 shows the risk analysis between sex, assault and any fall. Males sustained their injuries 2.2 times more likely because of assault than females (95% CI 1.5–3.0, *p* < 0.0001). Females sustained their injuries 1.5 times more likely because of any fall than males (95% CI 1.3–1.7, *p* < 0.0001).

**Table 5:** Calculation of risk ratio (RR) by primary outcomes between sexes.

Sex	Assault/yes		Assault/no		Total		RR (95% CI)	*P*
	*n*	%	*n*	%	*n*	%		
Male	355	41.4	503	58.6	858	84.6	2.2 (1.5–3.0)	<0.0001
Female	30	19.2	126	80.8	156	15.4	ref	
Total	385	38.0	629	62.0	1,014	100.0		
	Any fall/yes		Any fall/no		Total		RR (95% CI)	*P*
	***n***	**%**	***n***	**%**	***n***	**%**		
Male	336	39.2	522	60.8	858	84.6	ref	
Female	91	58.3	65	41.7	156	15.4	1.5 (1.3–1.7)	<0.0001
Total	427	42.1	587	57.9	1,014	100.0		

CI: confidence interval; ref: reference category.

Table 6 summarises the multivariable logistic regression analyses for assault and any fall. Significant predictors for assault were male sex and younger age. When adjusted with age, males had 2.8 times greater odds (95% CI 1.8–4.3, *p* < 0.001) for assault as compared to females. Patients aged ≤ 42.35 had 4.5 times greater odds (95% CI 3.4–6.0, *p* < 0.001) for assault than those who were older. Significant predictors for any fall were female sex and older age. When adjusted with age, females had 2.0 times greater odds (95% CI 1.4–2.9, *p* < 0.001) for any fall as compared to males. Patients aged ≥ 42.36 had 3.7 times greater odds (95% CI 2.8–4.8, *p* < 0.001) for any fall than those who were younger.

**Table 6:** Summary of multivariable logistic regression analysis for primary outcomes.

	Assault		Any fall	
	OR (95% CI)	*P*	OR (95% CI)	*P*
**Unadjusted**				
**Sex**				
Male	3.0 (1.9–4.5)	< 0.001	ref	
Female	ref		2.2 (1.5–3.1)	< 0.001
**Age group**				
≤ 42.35	4.5 (3.4–6.0)	< 0.001	ref	
≥ 42.36	ref		3.7 (2.8–4.8)	< 0.001
Adjusted*				
Male	2.8 (1.8–4.3)	< 0.001	ref	
Female	ref		2.0 (1.4–2.9)	< 0.001

OR: odds ratio; CI: confidence interval; ref: reference category.

* Adjusted with age.

Table 7 summarises the logistic regression analysis by secondary outcomes between the sexes. Females had 1.8 times greater odds (95% CI 1.3–2.6, *p* = 0.001) for fall at ground level compared to the odds of males. Males had 2.4 times greater odds (95% CI 1.1–5.1, p = 0.020) for isolated cranial fracture and 1.7 times greater odds (95% CI 1.1–2.6, p = 0.026) for FISS ≥ 3 compared to the odds of females. Regarding other secondary outcome variables, no statistically significant differences between sexes were observed.

**Table 7:** Univariable logistic regression analysis by secondary outcomes between sexes.

	Male	Female	*P*
	OR (95% CI)	OR (95% CI)	
Fall at ground level	ref	1.8 (1.3–2.6)	0.001
Fall in stairs	ref	1.4 (0.8–2.6)	0.252
Fall from height	ref	1.7 (0.8–3.4)	0.142
Bicycle	ref	1.6 (0.95–2.5)	0.077
Unknown	ref	1.4 (0.7–3.0)	0.382
Motor vehicle accident	3.1 (0.7–13.0)	ref	0.125
Struck by object	1.5 (0.2–11.8)	ref	0.723
High energy trauma mechanism	ref	1.3 (0.8–2.0)	0.268
Weekday (Monday–Thursday)	ref	1.1 (0.8–1.6)	0.588
Weekend (Friday–Sunday)	ref	0.9 (0.6–1.3)	0.588
Winter	1.1 (0.7–1.6)	ref	0.789
Spring	1.1 (0.7–1.7)	ref	0.569
Summer	0.7 (0.5–1.1)	ref	0.743
Fall	1.2 (0.8–1.8)	ref	0.353
Isolated facial fracture	0.7 (0.5–1.1)	ref	0.733
Isolated cranial fracture	2.4 (1.1–5.1)	ref	0.020
Combined facial and cranial fracture	0.9 (0.6–1.5)	ref	0.944
FISS ≤ 2	0.6 (0.4–0.9)	ref	0.026
FISS ≥ 3	1.7 (1.1–2.6)	ref	0.026

OR: odds ratio; CI: confidence interval; ref: reference category.

Isolated facial fracture: mandible/midface fracture; Isolated cranial fracture: frontal bone/orbital roof/skull vault/skull base fracture.

FISS: facial injury severity score, calculated in 893 patients with mandible, midfacial and/or upper facial third fractures.

## Discussion

The aim of the present study was to investigate the characteristics of patients having sustained craniofacial fractures under the influence of alcohol. The specific aims were to identify trauma mechanisms and types and severities of fractures, and to compare the frequencies of them between males and females.

Our results revealed that 35.5% of all adult patients with craniofacial fractures had sustained their injuries under the influence of alcohol, that assaults and falls were the most common reasons for alcohol-related injuries, and that males, by far, predominated among patients who had been intoxicated. Moreover, significant differences between males and females were observed: males had 2.8 times greater odds for assault, 2.4 times greater odds for isolated cranial fracture and 1.7 times greater odds for FISS ≥ 3 compared to the odds of females. Females had 2.0 times greater odds for any fall compared to the odds of males.

Retrospective cohort studies have shown that 18–37% of patients with facial fractures were verifiably under the influence of alcohol at the time of the injury [3,4,6–8,10], the corresponding rate in the present study being 35.5%. These rates are clearly lower than the one observed in a recently published prospective study by Hirvikangas et al. [9]. During a 1-year study period, the authors recruited altogether 166 adult patients with facial fractures, collecting data about patients’ alcohol use with the aid of a structured questionnaire and an additional interview. The rate of patients who were under the influence of alcohol at the time of the injury was as much as 55%. The differences in results highlight the main general drawback of retrospective studies with respect to reliability of patient file data. Some patients are likely not tested for breath or blood alcohol at admission, and in some, alcohol odour is perhaps not documented in the patient file even if identified. Moreover, if seeking treatment is delayed, information about intoxication status might be missing or false. As reported by Alvi et al. [3], alcohol status at the time of facial injury remained unknown in as much as 23.8% of injured patients. We assume that the 35.5% rate of intoxicated patients identified in the present study is, at least to some extent, an underestimation.

Previously published studies have shown that the vast majority of alcohol-related facial injuries occur in males, the male to female ratio having varied between 5.6:1 and 7:1 [7,9,14]. Also in the present study, the great majority were males (84.6%). The results reflect the differences in drinking habits between men and women on a general level: the prevalence of hazardous drinking among Finnish men is almost three times higher than among women, and the rate for men who are per definition either hazardous drinkers, alcohol abusers or alcohol dependents is 17.4% as compared to 5.0% in women [15]. Heavy alcohol consumption in general correlates with intoxication status at the time of the injury: the proportion of regular heavy drinkers was significantly higher among facial fracture patients who were under the influence of alcohol at the time of injury than among those who were not [9]. Although men predominated in the present study, the role of alcohol in women’s injuries are not to be diminished; our data on 156 women correspond to an average of 2.2 intoxicated women every month during the study period.

Notable rates (34–65%) of interpersonal violence among patients who sustained their injuries while under the influence of alcohol have previously been presented [7,14] and, vice versa, notable rates of alcohol intoxication at the time of the injury (55–84%) have been shown among patients who sustained their injuries because of interpersonal violence [5,9]. Similar results were observed in the present study; assault was frequent (38%), especially among men (41.4%). In association with violence in general, there exist some significant differences between men and women regarding the scene of the accident as well as the perpetrator. Men experience violence most often in restaurants, bars and other public areas, whereas women are more frequently assaulted in their homes [16,17]. Men are assaulted more frequently by an unknown perpetrator (46.9%) compared to women (24.8%), whereas the spouse or partner is more frequently the perpetrator where women are involved (45.7%) compared to men (5.4%) [18].

It has been estimated that of all female victims of assault, some 30–40% may be victims of domestic violence [16,19], with the rate being notably higher than among men (1%) [16]. Moreover, it has been estimated that 39% of homicides among women have been committed by intimate partners, with such violence being commonly the end result of a long history of abuse [20]. As described in the study conducted by Hackenberg et al. [21], the Töölö Hospital Emergency Department, in which the present study was executed, uses a structured form for patients who present as victims of violence. Despite this, we assume that the 19.2% rate of assaulted women observed in the present study is an underestimation: patients in general and women in particular seem to be reluctant to report partner abuse spontaneously. McLeer et al. [19] demonstrated that when a systematic protocol designed to recognise women who sustained injuries caused by battering were introduced, the rate of battered women identified by the emergency department staff increased significantly from 5.6 to 30.0%. Eight years later, when the protocol was not in use anymore, the rate of battered women identified in the same emergency department had decreased to 7.7%. If domestic violence is considered likely, and the patient gives an indeterminable anamnesis regarding the mechanism of injury or a history that does not correspond to the nature of the injuries, additional inquiries should be performed in order to intervene whenever needed.

In addition to exposure to violence, alcohol intoxication predisposes to various types of accidents. In the present study, a fall at ground level was the second most common mechanism of injury (29.9%). When all different types of falls were grouped together, ‘any fall’ remained the single most common aetiology (42.1%), especially among women. Statistics from another Finnish trauma hospital covering different types of injuries showed notable rates of intoxicated patients in association with falls on stairs (36%), bicycle accidents (31%), falls at ground level (22%) and motor vehicle accidents (16%) [22]. Increasing blood alcohol content causes loss of inhibitions, debilitates body control and coordination, decreases alertness and increases impulsiveness and risk-taking behaviour. In order to prevent further accidents, patients having sustained their injuries while intoxicated should be identified and motivated to stop or at least decrease binge drinking. Brief alcohol interventions should be executed as they have been proven efficient in reducing both alcohol intake and the risk for trauma recurrence [23].

Previously published studies have shown that intoxication by alcohol at the time of the accident predisposes to severe facial injuries. In a retrospective study from Australia [14], the authors compared 4,293 facial fracture patients in the alcohol group to 50,393 patients in the non-alcohol group. The patients in the alcohol group had multiple facial fractures more frequently (36%) than those in the non-alcohol group (25%). Moreover, alcohol involvement was associated with more complex patterns of fractures. In another Australian prospective study [24], 107 patients who were intoxicated by alcohol were compared to 95 who were not intoxicated. The authors observed that patients in the alcohol group had a significantly higher mean maxillofacial injury severity score than patients in the non-alcoholic group. In the present study, the great majority of intoxicated patients (72.2%) had a low FISS score of less than three, the explanation being likely that only 15.7% of the patients had been injured via high energy trauma mechanisms. However, the finding that males had an almost two times greater odds for FISS ≥ 3 indicate that the combination of alcohol intoxication and assault may predispose to severe facial fractures. Indeed, as shown by O’Meara et al. [24], patients whose facial fractures were the result of interpersonal violence had more severe injuries when alcohol was involved. Moreover, intoxicated patients who had been involved in interpersonal violence had a 1.44-fold risk for surgical intervention when compared to intoxicated patients who had sustained their injuries because of other trauma mechanisms.

The main limitation of retrospective studies is missing data. In the present study, information about the trauma mechanism was missing for only 44 patients (4.3%), either because of inadequate journal entries or because patients could not recall what had happened. However, because of the well-known fact that some patients withhold relevant information from doctors to avoid embarrassment, some of the trauma mechanisms in the present study may be incorrect. This might apply to a few patients who were physically abused or who presented late after alcohol-related injury.

## Conclusions

Particularly male patients are frequently under the influence of alcohol at the time of injury, predisposing them to assault and severe facial fractures more often than females. In order to prevent recurrent injuries, units treating these patients should have established practices for how to identify unhealthy alcohol habits, how to bring the topic to discussion and how to intervene.
